# Multiplex chromogenic immunohistochemistry to stain and analyze paraffin tissue sections from the mouse or human

**DOI:** 10.1016/j.xpro.2022.101879

**Published:** 2022-12-02

**Authors:** Oscar Maiques, Victoria Sanz-Moreno

**Affiliations:** 1 Barts Cancer Institute, Queen Mary University of London, John Vane Science Building, Charterhouse Square, London EC1M 6BQ, UK

**Keywords:** Cancer, High Throughput Screening, Immunology, Microscopy, Molecular/Chemical Probes

## Abstract

Here we describe a multiplex chromogenic immunohistochemistry platform to stain and analyze two markers in paraffin tissue sections from mouse or human. The basis of the protocol is a series of stripping and re-probing steps with subsequent image analysis, which allows the user to perform multiplex imaging in a reliable and affordable manner. Here, we describe specific usage to assess the levels of PD-L1 in tumor-associated macrophages. We have used different antibodies and assessed this protocol for up to five consecutive antibodies per slide.

For complete details on the use and execution of this protocol, please refer to Orgaz et al. (2020).^1^

## Highlights

• Chromogenic immunohistochemistry protocol that allows multiplexing
• Accessible and reliable protocol with multiple applications
• Multiplex protocol using brightfield microscopy
• Image processing and analysis simple and suitable for open-source softwares

## Abstract (editor highlights)

Publisher’s note: Undertaking any experimental protocol requires adherence to local institutional guidelines for laboratory safety and ethics.

## Graphical abstract

**Figure:**
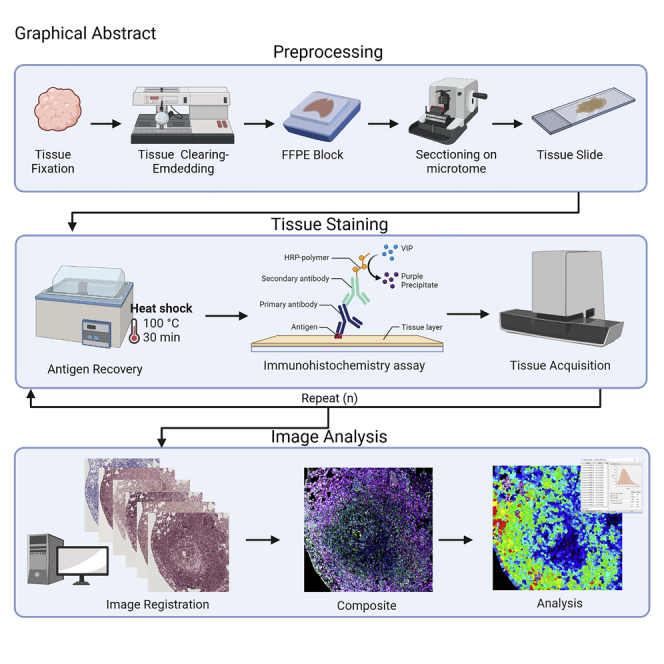

## Before you begin

This protocol below describes the specific steps for digital multiplex and image analysis in murine 5555 melanoma cell line grown subcutaneously in C57BL/6J mice. However, this protocol can be adapted to different antibodies and tissue types. We have also used this protocol in different tumor types (melanoma, breast cancer) and species (human and mouse).^2^^,^^3^

### Tissue collection: Establishment of 5555 tumors

**Timing: Depends on the study**

We describe multiplex IHC analysis in tumors derived from 5555-anti-PD1 non-responders generated in Orgaz JL, et al.^1^ but the same protocol can be applied to any mouse or human tissue.

### Preprocessing: Preparation of paraffin-embedded tissue sections

**Timing: 2–3 days**

1. Fix tissues with 10% (v/v) neutral buffered formalin (NBF) or 4% (w/v) formaldehyde solution (PFA) for 12–24 h at 4°C. Make sure enough fixative is used to cover tissues. Fixative volume should be 5–10 times the volume of tissue being fixed.

**CRITICAL:** Time to fixation is critical. Fixation after removal is strongly advised since tissue autolysis starts at this time. Tissues can be kept at 4°C in PBS plus Ca & Mg (100 mg/L, each, see key resources table) to slow down the process. Maximum time for this step is 4 h.

***Note:*** After fixation, tissues can be kept in 70% ethanol and stored at 4°C if there is no immediate need for paraffin embedding (if experiments are carried with different endpoints). Fixation for 14–15 h at 4°C is recommended while tissue size and room temperature can vary slightly. Formaldehyde penetrates tissues at 1 mm/h. Specimens should not exceed 1.5 × 1.5 × 0.4 cm.

2. Locate and orientate the fixed tissues in embedding cassettes as desired for the experiment.

***Note:*** For example, primary melanoma tumors used to study local invasion are cut in half vertically to characterize the transition from tumor body to invasive front under the microscope.

3. Process for paraffin embedding schedule as follows (total 16 h):
  ***Note:*** 600 mL glass container can be used (see key resources table).
  a. 70% Ethanol, 2 × 1 h.
  b. 80% Ethanol, 1 h.
  c. 95% Ethanol, 1 h.
  d. 100% Ethanol, 3 × 1.5 h.
  e. Xylene 3 × 1.5 h.
  f. Paraffin wax (58°C–60°C), 2 × 2 h.
  g. Embedding tissues into paraffin blocks.
    **CRITICAL:** All work involving ethanol and xylene must be performed under a fume hood.
    ***Alternatives:*** Research Institutions usually process this step via their Pathology Facilities where the protocol is standardized and monitored using tissue processors.

4. Trim paraffin blocks as necessary and cut at 4–5 μm using the microtome.

**CRITICAL:** Sections should be thinner than 5 μm to ensure antibodies penetrate the tissue.

5. Place paraffin ribbon in water bath at about 35°C–40°C, to allow tissue to expand.
6. Mount sections onto slides. Use slides to pick the sections out of the water bath and store upright in a slide rack.
7. Allow sections to air dry for 14–15 h at 21°C–25°C. Afterwards, place the slides in the oven at 60°C for 1 h.

***Note:*** Tissue slides can be kept at 21°C–25°C for no longer than 3 months.^4^ Antigenicity could be affected after longer periods of time.

### Institutional permissions

This research complies with all relevant ethical regulations; all animals were maintained under specific pathogen-free conditions and handled in accordance with the Institutional Committees on Animal Welfare of the UK Home Office (The Home Office Animals Scientific Procedures Act, 1986). All animal experiments were approved by the Ethical Review Process Committees at Barts Cancer Institute or King’s College London and carried out under license from the Home Office, UK.

## Key resources table

**Table:** 

REAGENT or RESOURCE	SOURCE	IDENTIFIER
**Antibodies**		

CD206	Abcam	Cat# ab64693; RRID: AB_1523910
PD-L1 clone E1L3N	Cell Signaling Technology	Cat# 13684; RRID: AB_2687655
AffiniPure Fab Fragment Goat Anti-Rabbit IgG (H+L)	Jackson Immunology	Cat# 111-007-003

**Chemicals, peptides, and recombinant proteins**		

Emerald antibody diluent	Sigma-Aldrich	Cat# 936B-08
Antigen unmasking solution, citrate buffer (100×)	Vector Laboratories	Cat# H-3300
ImmEdge Hydrophobic Barrier PAP Pen	Vector Laboratories	Cat# H-4000
ImmPress Polymer Reagent anti-Rabbit	Vector Laboratories	Cat# MP-7451-15
VIP HRP substrate chromogen	Vector Laboratories	Cat# SK-4600
DPX mounting media	Sigma-Aldrich	Cat# 6522
Harris hematoxylin	Sigma-Aldrich	Cat# HHS16
Trizma base	Sigma-Aldrich	Cat# T1503
NaCl	Sigma-Aldrich	Cat# S9888
Tween 20	Sigma-Aldrich	Cat# P7949
HCL		
Hydrogen peroxide 30%	VWR	Cat# 23619.264
PBS (phosphate-buffered saline) (pH 7.4)	Gibco	Cat# 10010023
DPBS, calcium, magnesium	Gibco	Cat #14040117
Ethanol absolute (99%)	Fisher Scientific Uk Ltd	Cat# 10437341
Ammonium hydroxide	Sigma-Aldrich	Cat# 221228-1L-A
Xylene	Fisher Scientific Uk Ltd	Cat# 10385910
10% neutral buffered formalin	CellPath Plc	Cat # BAF-0100-25A
Dako wash buffer 10×	Agilent	Cat # S300685-2

**Other**		

NanoZoomer S210 Digital slide scanner	Hamamatsu	Cat# C13239-01
Tefal 6 Liter stainless steel pressure cooker	Tefal	Cat # 3045384362433
Hot plate	Cole-Parmer	Cat# 11476508
Microtome	Leica	Cat# RM2125 RTS
Slide jars	Simport	Cat# M906-12AS
Cover slides	VWR	Cat# 631-0146
IHC slides	Thermo Fisher	Cat# 12312148
Embedding cassettes	Epredia	Cat# 16323986
RS PRO Borosilicate Glass 600 mL Beaker	RS PRO	Cat# 461-1137
Fume hood	Fumair	Cat# 52716
Staining tray	Agar Scientific	Cat# AGAR_L4474
Oven (hybridizer hb-1)	Techne	Cat# 39455-03

**Software and algorithms**		

ImageJ FIJI	https://imagej.nih.gov/ij/https://fiji.sc/	1.53f51
QuPath	https://qupath.github.io/	0.2.0m.8
NDP.view2	https://www.hamamatsu.com/us/en/product/type/U12388-01/index.html	2.7.25
GraphPad	https://www.graphpad.com/scientific-software/prism/	9.0.2

## Materials and equipment

**Table:** H_2_O_2_ endogenous peroxidase blocking solution

Reagent	Final concentration	Amount
Hydrogen peroxide 30%	2%	40 mL
Ethanol Absolute (99%)	97%	560 mL
**Total**	**N/A**	**600 mL**

***Note:*** Hydrogen peroxide 30% stock bottle is stored at RT. Once prepared fill in two staining slide jars at RT under the fume hood.

**Table:** Citrate antigen retrieval

Reagent	Final concentration	Amount
Citrate buffer (100×)	1×	20 mL
ddH_2_O	N/A	1,980 mL
**Total**	**N/A**	**2,000 mL**

**Table:** 10× TBS-Tween 20 – IHC wash buffer

Reagent	Final concentration	Amount
Trizma base	0.5 M	61 g
NaCl	9%	90 g
Tween 20	0.1%	1 mL
ddH_2_O	N/A	999 mL
**Total**	**N/A**	**1,000 mL**

***Note:*** Mix to dissolve and adjust pH to 8.4 using concentrated HCl and then add 1 mL Tween 20.

Store this solution at RT. Dilute 1:10 with distilled water before use and adjust pH if necessary.

***Alternatives:*** Can be used commercial wash buffer (10× Dako Wash Buffer, Agilent).

**Table:** Acid alcohol (1%)

Reagent	Final concentration	Amount
HCL	1%	5 mL
Ethanol Absolute (99%)	70%	350 mL
ddH_2_O	N/A	145 mL
**Total**	**N/A**	**500 mL**

**Table:** Ammonia water solution (0.2%)

Reagent	Final concentration	Amount
Ammonium hydroxide (concentrated)	0.2%	2 mL
ddH_2_O	N/A	998 mL
**Total**	**N/A**	**1,000 mL**

**Table:** VIP HRP substrate chromogen

Reagent	Final concentration	Amount
Reagent 1	N/A	3 drops
Reagent 2	N/A	3 drops
Reagent 3	N/A	3 drops
Reagent 4	N/A	3 drops
PBS	N/A	5 mL
**Total**	**N/A**	∼**5 mL**

***Note:*** Table above shows instructions as per manufacturer indications. 5 mL is the minimum volume recommended. https://vectorlabs.com/products/substrates/vector-vip-hrp-substrate-kit.

## Step-by-step method details

### Rehydration and antigen retrieval round 1

**Timing: 1–2 h**

**Figure 1:**
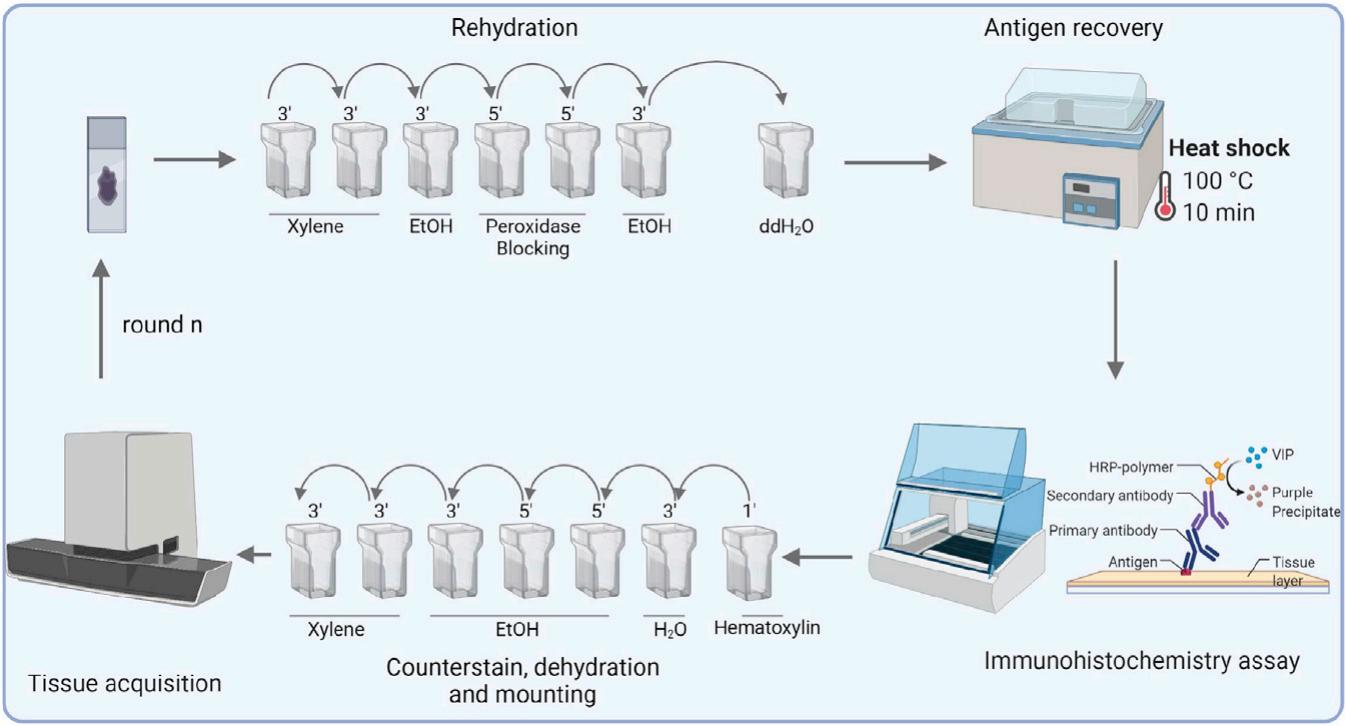
Schematic of cyclic rounds of staining and stripping Staining starts in rehydration and endogenous peroxidase blocking. Continues with heat-induced antigen retrieval process with a pH solution. Staining steps with primary and secondary antibodies follow. The secondary antibody is coupled to HRP-enzyme, which in the presence of VIP substrate precipitates in a chromogenic reaction (purple). Staining finishes with counterstain, dehydration and mounting of cover slip. Finally, the staining is imaged using a microscope slide scanner.

This step describes how to rehydrate tissue sections and perform antigen retrieval using a pressure cooker (See Figure 1).

1. Make sure slides are marked with appropriate information.

***Note:*** Pencil is recommended for labelling the slides. Pens can be washed away due to subsequent steps using alcohols. Alternatively, pathology services use histology printer slides.

2. Load slides into a rack and place in de-waxing xylenes, 2 × 5 min.
3. Transfer slides to ethanol 99% solution for 3 min.
4. Block any endogenous peroxidase activity by placing slides into H_2_O_2_ Endogenous peroxidase blocking solution, 2 × 3 min.

***Note:*** See recipe in materials and equipment section “H_2_O_2_ Endogenous peroxidase blocking solution”.

5. Transfer to final ethanol 99% solution for 3 min.

***Alternatives:*** Gradual rehydration with decreasing concentrations of ethanol (99%, 70% and 50%) leads to comparable results.

6. Rinse in running water, 1 min.

7. Use pressure cooker for heat-induced antigen retrieval (HIAR).
  ***Alternatives:*** A water bath or microwave are also reliable methods for HIAR.
  a. Switch on hotplate and set to full temperature (400°C) by turning the dial to maximum.
    **CRITICAL:** For safety reasons, this protocol has been adapted for a conventional pressure cooker with a laboratory hot plate. Electrical pressure cookers can also be used.
  b. Prepare the Antigen-retrieval buffer. See recipe in materials and equipment section “Citrate antigen retrieval”.
  c. Replace the lid, but do not lock.
  d. Allow solution to warm until boiling (∼10 min).
  e. Carefully lower the rehydrated histological slides into the boiling solution.
  f. Replace lid and lock and set pressure selector to the highest-pressure symbol.
  g. Once the red pressure indicator has risen and a steady flow of steam is escaping from the outlet valve, start timing for 10 min.
  h. After 10 min, switch off the hotplate using the dial, carefully remove the pressure cooker and transfer to a sink.
    ***Note:*** Changes in concentration, pH and Formalin exposure will introduce variability. Some epitopes are formalin-resistant while others undergo substantial changes (formalin-sensitive).
    ***Alternatives:*** Citrate buffer (pH 6.0) is the most used solution for HIAR. This protocol uses citric buffer for antigen retrieval. Alternatively, EDTA (pH 8.0), Tris-EDTA (pH 9.0), and Tris (pH 10.0) buffers can be used. The pH of the buffers is an essential factor for antigen refolding or for antigenic determinant exposure. pH 8–9 is suitable for most antigens, although certain nuclear antigens show optimal staining at pH.^5^ Unless the antigen retrieval method is stated on the antibody datasheet, the optimal method for each antigen must be tested experimentally.
    **CRITICAL:** Enzymatic digestion such as proteinase K, trypsin and pepsin are not suitable for the current IHC multiplex protocol. Enzymatic digestion compromises the detection of epitopes that are suitable for HIAR. For instance, proteinase K is recommended when using anti-F4/80 antibodies, while poor antigen quality after consecutive anti-CD206 staining using pressure cooker antigen retrieval will lead to negative staining.

8. Lifting (or removing) the valve, pushing a button or turning a dial (depending on the pressure cooker). This method involves strong vapor evacuation.

**CRITICAL:** This release method does not cool down the pressure cooker like the cold-water release method. To avoid scalding injury, care must be taken when releasing the steam.

9. Once pressure cooker is open, allow the tissue slides to cool down in running tap water for 5 min.
10. Take out the slides from the pressure cooker and place them in a slide jar with ddH_2_0 ensuring the slides do not dry. The slides are now ready for subsequent staining.

***Note:*** Tissue slides can be kept in water during 1–2 h. However, if longer periods are required, slides should be kept in TBS-Tween 20 (TBST).

**CRITICAL:** Reagents for dehydration and rehydration inside the staining jars can be kept under the fume hood at RT. However, it is important to change them once a week especially the H_2_O_2_ endogenous peroxidase blocking solution.

### Immunohistochemistry round 1

**Timing: 3 h**

This step describes how to detect antigen-antibody binding in tissue sections (Figure 1).

***Note:*** All the incubation processes should be performed under a humidified chamber in a staining tray at RT and covered to avoid slides drying out. To create a humidified chamber, add a piece of tissue paper covered in water at the bottom of the tray. Washes can be performed in histology staining jars.

11. Use ImmEdge Hydrophobic Barrier PAP Pen to draw a circle around a specimen on the slide. It will help to keep reagents localized on tissue specimens and prevents mixing of reagents when multiple sections are mounted on the same slide.

**CRITICAL:** PAP pen is considered as Cancer and Reproductive Harm. www.P65Warnings.ca.gov.

12. Transfer the slides into TBST.

***Note:*** see recipe in materials and equipment section.

***Alternatives:*** Commercial wash buffers with similar composition such as Dako wash buffer 10× can be used instead.

13. Remove unspecific staining by incubating the sections with 1%BSA-TBS for 30 min RT.

***Alternatives:*** Normal serum can be used to block non-specific binding matching the species of the secondary antibody. Serum contains antibodies that bind to non-specific sites in the tissue or to Fc receptors. Pooled serum from healthy adult animals at 2.5% is commercially available. In this experiment, 2.5% Normal Goat Serum was used since the secondary antibody was raised in Goat.

14. Add 100–300 μL (depending on size of specimen) of primary antibody, in this case Anti-PD-L1 (1:200, 4.37 μm/mL) to antibody diluent (Emerald antibody diluent) for 40 min RT.

***Alternatives:*** Emerald diluent is commercially available –but 1% BSA-TBS can be used instead.

**CRITICAL:** This protocol was optimized for PD-L1 and CD206 detection in consecutive rounds. The antibody order needs to be optimized for each experiment.

15. Rinse with TBST for 2 × 5 min at RT.
16. Apply 100–300 μL (depending on specimen size) anti-rabbit secondary antibody polymer conjugated (ImmPRESS® Polymer Reagent) Ready to Use (RTU). Incubate for 45 min at RT.

***Note:*** ImmPRESS reagents are sold as RTU solutions. Small biopsies or tissue sections such as mouse spleen are covered in 100 μL. However larger sections such as those from mouse primary tumors may require 300 μL to cover the entire section.

17. Rinse with TBST for 2 × 5 min at RT.

***Note:*** Meanwhile prepare VIP substrate chromogen. Follow manufacturer’s instructions for preparation (3 drops each bottle in 5 mL of PBS).

See link: https://vectorlabs.com/products/substrates/vector-vip-hrp-substrate-kit.

**CRITICAL:** It is especially important to use PBS as diluent for VIP chromogen. ddH2O, antibody diluent or 1%BSA-TBS are not recommended. As specified by the manufacturer instructions, deionized water may contain inhibitors of the peroxidase reaction. Solutions containing sodium azide or other inhibitors of peroxidase activity should not be used when diluting the peroxidase substrate.

18. Apply Vector VIP HRP substrate chromogen for 10 min at RT.

***Note:*** Previous published protocols use alcohol soluble chromogens for multiplex staining. VIP chromogen is stable in alcohol, but it is heat sensitive. Advantages of this chromogen: it is as sensitive as DAB and can be removed using regular antigen retrieval procedures such as pressure cooker, water bath or microwave. See troubleshooting section, problem 1.

19. Transfer slides to running tap water for 2 min at RT.

**CRITICAL:** Avoid direct contact from running tap water, it could displace the tissue attached to the glass slide.

**CRITICAL:** Always run appropriate positive and negative control sections with each staining batch. For the purposes of this protocol mouse spleen was used as positive control.

***Note:*** Tissues where presence of a specific cellular population or antigen are known or expected can be used as positive controls in IHC. For unknown or undescribed antigens of interest https://www.proteinatlas.org/ can be an indicator. For immune cell populations in mice, mouse spleens are commonly used as positive controls. For human samples, tonsils are used due to easy access for pathology units. Appendix is also a widely used control since it is a tissue rich in inflammatory cells, epithelial cells and stroma. Negative controls are also recommended (no primary antibody or Isotype control antibody can be used). To evaluate specificity of new antibodies, cell lines transfected with siRNA targeting the gene/protein of interest can be used. Agar pellets of cells after RNAi transfections are embedded in paraffin and the same IHC protocol can be applied to confirm specific antibody binding. Efficiency of RNAi transfection needs to be confirmed by either qPCR or western blot.

### Counterstain, clear, and mount round 1

**Timing: 30 min**

This step describes how to counterstain with Hematoxylin, dehydrate the tissues and mount with cover-slides (Figure 1).

20. Incubate the slides in Harris Hematoxylin for 3 min at RT.
21. Transfer slides to running tap water for 2 min at RT.
22. Dip quickly in Acid alcohol (1%) for 2–3 s at RT.

**CRITICAL:** It is especially important not to leave the slides for longer than a few seconds in Acid alcohol, as it can remove most of the Hematoxylin and part of the VIP chromogen.

23. Transfer slides to running tap water in tap water for 2 min.
24. Incubate in Ammonia water solution (0.2%) for 3 min.
25. Rinse in tap water for 2 min.
26. Transfer slides to ethanol 99% solution for 3 × 3 min.

***Alternatives:*** Gradual dehydration with increasing concentrations of ethanol (50%, 70% and 99%) leads to comparable results.

27. Transfer slides to Xylene 2 × 3 min.
28. Mount the slides using DPX mounting media adding the coverslip.

**CRITICAL:** Aqueous mounting media is not suitable for dehydrated slides in Xylene or organic solvents.

29. Let to dry out the slides for 12–16 h under the fume hood.

***Alternatives:*** Gill’s or Meyer’s Hematoxylin can also be used. However, these hematoxylins are progressive, and steps 22–25 should be avoided. The incubation time should be 3–4 min longer if Gill’s or Meyer’s Hematoxylin are used.

### Tissue scanning round 1

**Timing: 30 min–4 h**

This step describes how to image the stained sections using a high-capacity digital pathology slide scanner. In this case, we have used NanoZoomer s210 Digital slide scanner from Hamamatsu (Figure 1).

30. Place the slides in the scanning tray and perform single or batch scanning, according to the number of slides in the experiment.
31. Set the Region of scan and place crosses in the focus points. Set 40× as resolution.

**Pause point:** If needed the slides can be stored and re-start Round 2 when desired. They can be used up to 6 times.

***Note:*** Mounted stained tissue slides should be stored at RT in an airtight container. Slides can be reused even after two years, if the slides are kept dry.

### Rehydration and antigen retrieval round 2

**Timing: 1 h**

This step describes how to perform rehydration and antigen retrieval procedure in slides that have already been stained (Figure 1).

32. Place the slides from round 1 into Xylene jar for at least 12–24 h.
33. Remove the coverslip carefully.
34. Re-start the process of rehydration and antigen retrieval as in “rehydration and antigen retrieval round 1” section.

**CRITICAL:** After antigen retrieval, slides should be unstained. If strong staining is observed in previous round, longer HIER is required to remove completely the VIP chromogen. This should be optimized by the user depending on the antibodies used. See troubleshooting section, problem 2.

### Immunohistochemistry round 2

**Timing: 4 h**

This step describes how to perform rehydration and antigen retrieval in slides that have been previously stained, while blocking the previous primary antibody.

35. Use PAP pen to draw a circle around a specimen on a slide.
36. Transfer the slides into TSBT.
37. Incubate sections for 30 min with Fab fragment anti-rabbit (1:50, diluted in TBS) (Jackson ImmunoResearch) for 30 min.

***Note:*** Step 37 might be optional depending on the species of the primary antibodies. For instance, if in round 1 the primary antibody was rabbit, and in round 2 is mouse or rat there is no need to use Fab.

38. Incubate sections for 40 min at RT with a solution of 100–300 μL (depending on size of specimen) of primary antibody, in this case anti-CD206 (1:1000, 1 μm/mL) in antibody diluent.
39. Rinse with TBST buffer for 2 × 5 min.
40. Apply 2–6 drops (depending on size of specimen) of secondary antibody polymer conjugated (ImmPRESS® Polymer Reagent) Ready to Use. Incubate for 45 min at RT. In this case it was an anti-rabbit antibody.
41. Rinse with TBST for 2 × 5 min. Meanwhile prepare VIP substrate chromogen. Follow manufacturer instructions for preparation, see recipe in materials and equipment section “VIP HRP substrate chromogen”.
42. Apply Vector VIP HRP substrate chromogen for 10 min at RT.

***Note:*** See troubleshooting section, problem 3.

43. Transfer slides to running tap water.

### Counterstain, clear, and mount round 2

**Timing: 30 min**

This part of the protocol is the same as Round 1 (Figure 1).

### Tissue scanning round 2

**Timing: 30 min to 4 h**

This part of the protocol is the same as Round 1 (Figure 1).

### Image alignment and pseudocolor image

**Timing: ∼30 min per slide**

**Figure 2:**
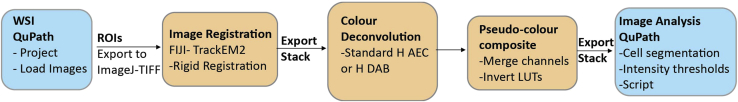
Workflow for image processing and image analysis using Fiji-ImageJ and Qupath

This step describes how to align images of two consecutive staining procedures and scans in the same tissue section. Furthermore, it creates a digital multiplex image that can be exported for quantification. To do so, QuPath^6^ and FIJI ImageJ^7^ were used (see Figure 2).

**Figure 3:**
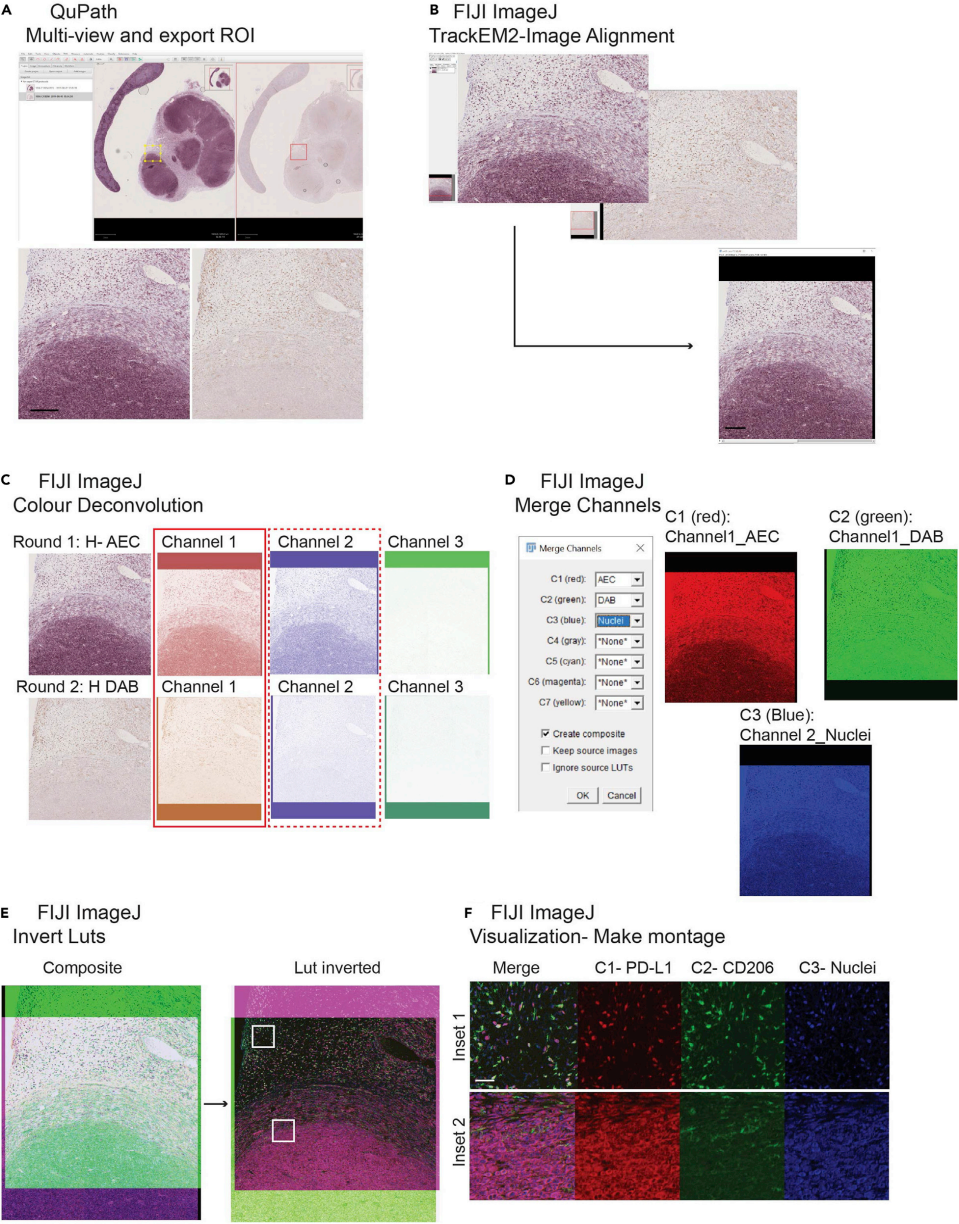
Generation of digital multiplex image Figure shows the different steps to create a composite image from different individual staining steps. (A) Representative image of multi-view in Qupath with the two staining steps. Images from the same section of tissue have been superimposed and the ROIs exported to ImageJ. (B) (Top) Representative image of the TrackEM2 tool in ImageJ for image registration before stack alignment. (Bottom) Exported stack after image co-registration. (C) Representative image of color deconvolution tool in ImageJ for the two co-registered ROIs. Round1 staining uses H AEC channels and Round 2 H DAB channels. (D) Representative image of Merge channel tool in ImageJ combining the Nuclei staining, Round1_Channel1 staining (CD206), and Round2_Channel2 staining (PD-L1). (E) Representative merged image (left) and with Lut inverted (right) creating a pseudo-colored image. (F) Representative image of the montage in two different insets for each of the different markers. Scale bar (A–E): 200 μm, (F): 50 μm.
47. Select similar ROIs in both images and export to ImageJ.
  a. Create Annotation in left image.
  b. Copy Annotation (Ctrl+D) and Paste in the other slide (SHIFT+E).
  c. Click ImageJ Icon>set resolution 1>OK.
  d. Repeat the process for all the annotations and save them in TIFF in ImageJ.

***Note:*** Store all the images in the same folder.

44. Create a project: Open QuPath and create a project with the images that need to be aligned.
  a. File> Project> Create a Project. Next, create a folder.
45. Adding images to the project.
  a. Press Add Images> Import images to project.
  b. Set image type as – Brightfield (H-DAB) and find the location of the images in “Choose files”. “Import” the images.
46. Set multi-view in QuPath to select the same region of interest (ROIs) in both images at the same time.
  a. “Right click”> “multi-view”>” Add column”. Next click on the image you want to add (Figure 3A).

48. Image alignment of the exported ROIs.
  a. Open FIJI imageJ>File>New>TrakEM2 (blank).
  b. Create a folder where you will move the images you need to align (Figure 3B).

***Note:*** Whole slide images (WSI) are compressed in pyramidal stacks. Registration of WSI is complex. TrakEM2 does not allow registration of WSI.

49. Importing ROIs for alignment.
  a. Drag the folder (should start with trackem2.) with the two images, in this case PD-L1 and CD206 from the same region to the TrakEM2 plug-in.
50. Set up the alignment tools. Once the folder containing the images is dragged, the plug-in will ask type of Directory.
  a. Press stack. Next, the slide separation by default 1.
  b. Next, “Right click” in the first image “Align”> “Align stack slices”. Leave by default all the Registration options (Rigid Registration).

***Note:*** Scroll the mouse to verify the alignment between the images. If images are not automatically aligned, manual supervised co-registration is possible. See troubleshooting section, problem 4.

51. Exporting aligned images.
  a. Press “Right click” on the image “Export”> ”Make flat image…”.
  b. Next choose Type: “RGB Color”, Start: “1: z=0.0 [layer]” and End: “2: z=1.0 [layer]”. Click Best Quality and Continue.
  c. Merged imaged in Tiff format will be created. Scroll the mouse to verify that the two staining images are completely aligned.
52. Transform stack to flat images.
  a. Go to “Image”> “Stacks”> “Stacks to Images”.
  b. Name is each registered image. For example, PD-L1, CD206 in this case.
53. Color Deconvolution. IHC images in this case have been counterstained with hematoxylin to identify the nucleus and perform a better alignment. Each image needs to be split in three channels: Channel 1: Nuclei or Hematoxylin, Channel 2: Chromogen VIP and Channel 3: Residual.
  a. Go to “Image”> “Color”> “Color Deconvolution”. We choose Vector: “H AEC” for PD-L1 and “H DAB” for CD206in the window (Figure 3C). AEC is a Red/Purple chromogen. Discard Channel 3 stack images.
  b. Repeat the process for Channel 1 and Channel 2 Stacks.
  c. Choose only one image for Channel1 or nuclear staining. Keep both images from Channel 2. Change the name to images to better identification. “Click” on the image “Image”> “Rename…” Give names such as Nuclei, PD-L1 and CD206.

***Note:*** Custom color deconvolution can be performed if the chromogens used are different to the ones used in the present protocol. See troubleshooting section, problem 5.

54. Merge the images.
  a. Go to “Image” > “Color”> “Merge Channels…” Choose for each image one color. For instance, in C1 (red) select “CD206” Image, in C2 (green) select “PD-L1” and in C3 (blue) select “Nuclei” (Figure 3D).
55. Create a pseudo-color composite image. Following the previous process, a new stack image will be generated.
  a. Go to “Image”> “Color”> “Channel Tool…” Channel to “Color” mode. For “Channel 1” go to “More”, select color “Red”, press “More” again and “Edit LUT” and “Invert…”
  b. Perform the same step in the Channel 2 and 3 using color Green and Blue accordingly.
  c. Finally Change “Color” mode to “Composite”. Save the image in Tiff format (Figure 3E and 3F).

### Image analysis and quantification

**Timing: 1–2 h for project**

**Figure 4:**
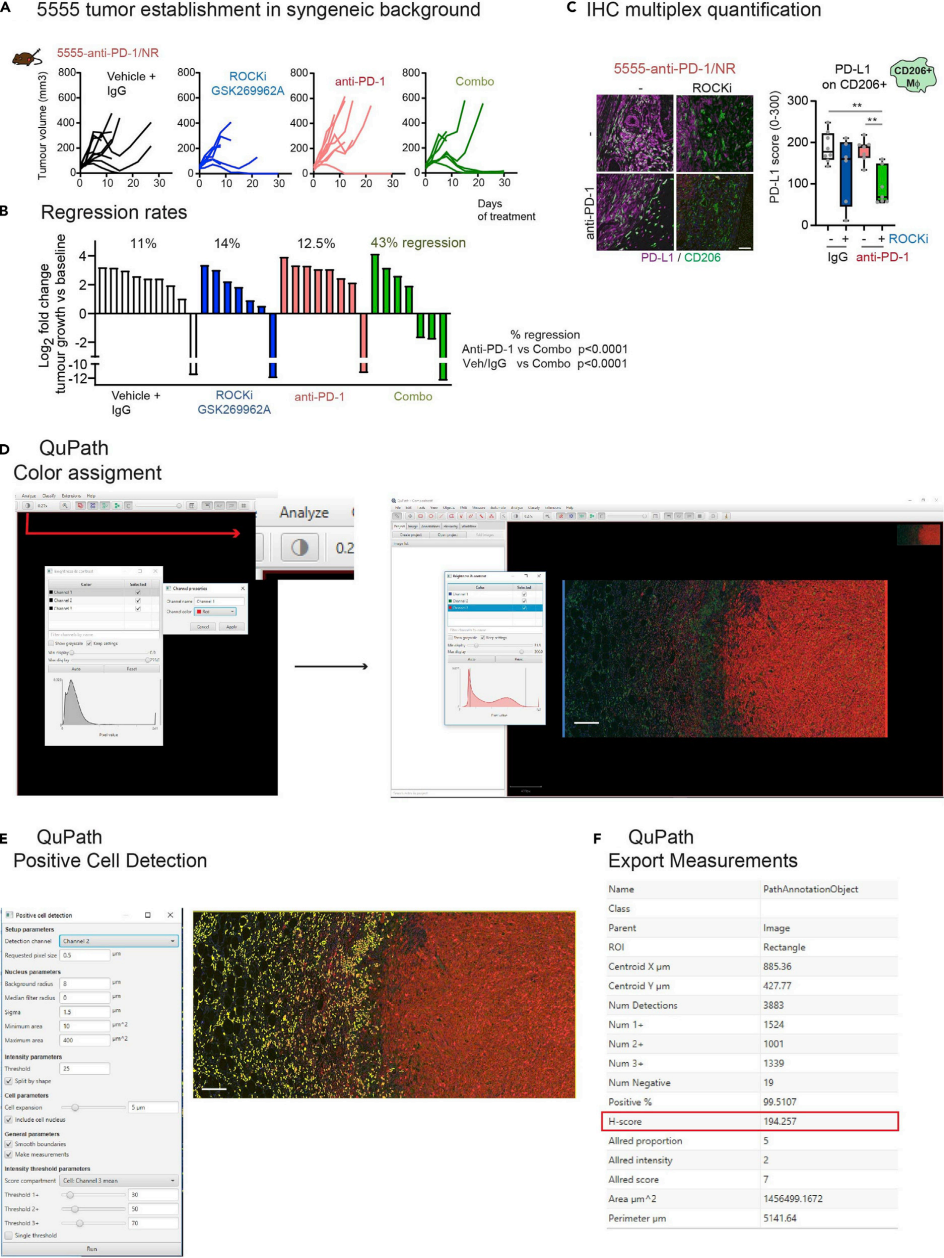
Image analysis of multiplexed images Steps described in image analysis and quantification section. (A–C) (A) Growth curves, (B) regression rates and (C) quantification of PD-L1 in CD206 positive cells from JL Orgaz., et al. Cancer Cell 2020.^1^ (D) Representative image showing how to change to Immunofluorescence settings and change color if convenient. (E) Representative image showing cell segmentation of CD206 positive cells and intensity values. (F) Example of measurements obtained after Positive cell detection analysis. Scale bar (C): 50 μm, (D and E): 150 μm.

In the example below, 5555 murine melanoma cells derived from the BRAF-V600E mouse melanoma model^8^ were selected for their lack of response to anti-PD-1 *in vivo* (anti-PD-1/NR). 1 million 5555-anti-PD-1/NR cells were injected subcutaneously into 7-week-old C57BL/6J female mice. Tumors were grown for up to 27 days (Figure 4A). Tumors were excised and fixed. The combination therapy of anti-PD1 plus ROCK inhibitor resulted in higher number of regressions (Figure 4B), and IHC was performed to assess effects on macrophage markers (Figure 4C).

***Note:*** Due to strict limits for tumor volume in the UK animal license animals were culled when tumor volumes reached maximum size allowed or before any signs of skin ulceration were detected (redness, glossy skin).

This step describes how to analyze images using QuPath of the ROIs alignment and process them using ImageJ (see Figure 4D–4F).

56. Create a project on Qupath.
57. Import the composite images from all the cases.
58. Change image type to “Fluorescence”

***Note:*** When importing the composite image (Figure 4D) you need to assign the colors manually. “Click” in brightness and contrast icon and add a color for each channel manually. In our example, we used Channel 1: Blue, Channel 2: Green and Channel 3: Red.

59. Create annotations in all the cases.

**CRITICAL:** Before any segmentation or analysis, you need to generate annotation in the region of interest.

60. Run> Cell detection (Figure 4E). Use “channel 2” (green, CD206) for detection channel. Furthermore, set-up an appropriate threshold to calculate different intensities. We used “Channel 3: Cell Mean” (red, PD-L1) for “score compartment” and threshold +1: 30, threshold +2: 50 and threshold +3:70.

***Note:*** Depending on antigen of interest subcellular localization, the score compartment should be optimized (Cell, Cytoplasm or Nuclear). Intensity thresholds should be adapted to the specific staining. Percentage of positivity instead of Histoscore can also be used as a measure by clicking “single threshold”.

***Note:*** For quantification purposes we use Histoscore instead of positive percentage since it provides semiquantitative information. Histoscore or H-score is used commonly in quantitative pathology. Hscore is calculated combining the intensity of staining (graded as: 0, non-staining; 1, weak; 2, median; or 3, strong) and the percentage of positive cells. The range of scores is from 0 to 300.

**CRITICAL:** Default settings may allow cell detection, but this step may require some optimization. See troubleshooting section, problem 6.

61. Export “Annotation measurement” table (Figure 4F).
62. Plot the H-score results. This method follows the analysis used in J.Orgaz et al. Cancer Cell 2020. We used Box-plot graph Boxplots show median (center line); interquartile range (box); min-max (whiskers); and individual mice (circles). p-values by ANOVA with Yekutieli correction (Figure 4C).

### Automation and batch analysis

Scripts can be used for Color deconvolution in FIJI and Image analysis in QuPath.

**Figure 5:**
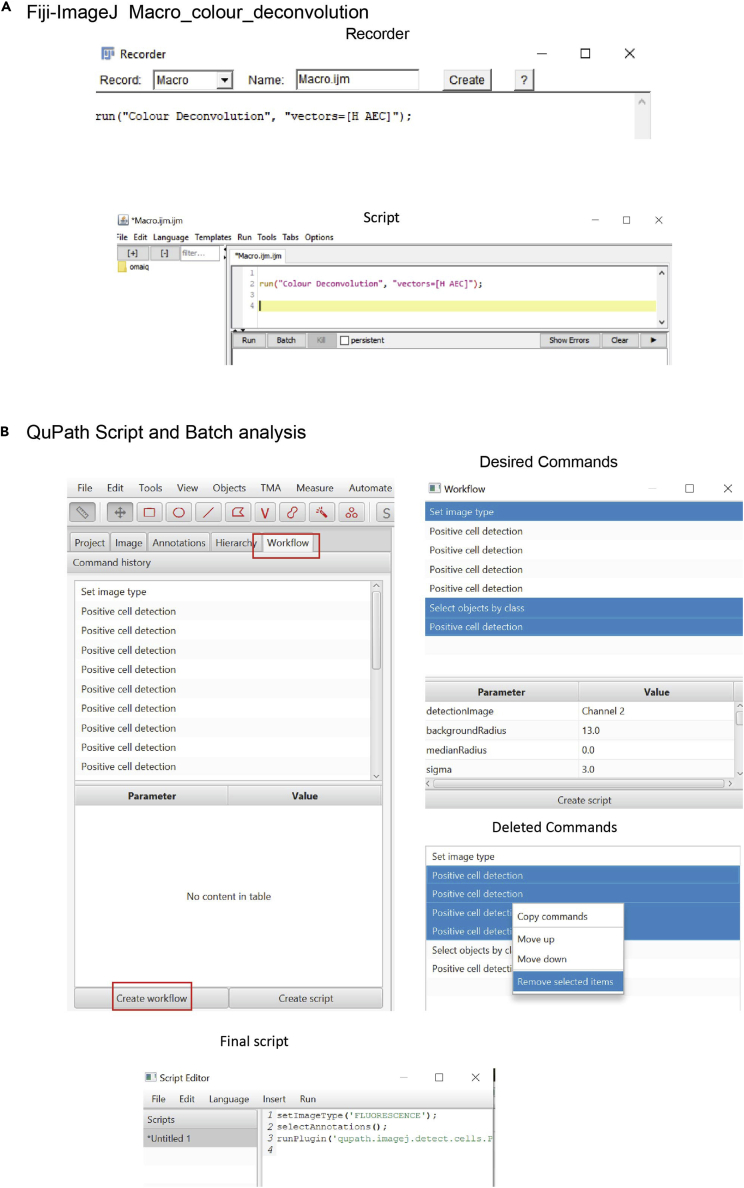
Automation and batch analysis (A) Schematic of a representative color deconvolution macro created in Fiji ImageJ. (B) Script and Batch analysis workflow in Qupath.
65. Use a control image as template for optimizing cell segmentation and intensity thresholds. Once optimized go to “Workflow”>” Create Workflow” and Select/Delete the desired command. In this case “Set Image type”, “Select objects by class”, and “Positive Cell Detection”.

***Note:*** Commands are ordered by chronological order. If the last Positive Cell Detection is the definitive, remove all previous commands (see Figure 5B).

63. Creating macros in Fiji-ImageJ. Use recording tool in macros section. Go to Plugins>Macros>Record.
64. Color Deconvolution Macro. Now that ImageJ is recording. Select the image of interest and perform color deconvolution. Go to the window macro and create a script (See Figure 5A).
  a. run (“Color Deconvolution”, “vectors= [H AEC]");
    Qupath allows automate analysis of a series of images. Creating scripts is user friendly using the Workflow Tab.

66. Create script. Store it and run it for the entire project. For this analysis the script is the following:
  setImageType(‘FLUORESCENCE');
  selectAnnotations();
  runPlugin(‘qupath.imagej.detect.cells.PositiveCellDetection’, '{"detectionImage”: “Channel 2″, “backgroundRadius”: 13.0, “medianRadius”: 0.0, “sigma”: 3.0, “minArea”: 30.0, “maxArea”: 1000.0, “threshold”: 32.0, “watershedPostProcess”: true, “cellExpansion”: 5.0, “includeNuclei”: true, “smoothBoundaries”: true, “makeMeasurements”: false, “thresholdCompartment”: “Cell: Channel 3 mean”, “thresholdPositive1”: 30.0, “thresholdPositive2”: 50.0, “thresholdPositive3”: 70.0, “singleThreshold”: false}');

## Expected outcomes

After performing stripping and re-reprobing and following the sections “image alignment and pseudocolor image” the user will be able to visualize the image as shown in Figure 3F. Merged images are uploaded in Qupath as described in “image analysis and quantification”. After following the steps provides the user should be able to obtain quantitative information regarding the.

## Limitations

Even if this is a robust, affordable and accessible pipeline, this protocol has several limitations: 1) Time consuming. Every marker has to be stained separately, which takes in average 6 h for staining plus scanning (time depends on the number of samples). Therefore, this protocol takes longer in comparison with simultaneous staining of several markers. A full protocol multiplexing 4–5 antibodies can take up to a week. However, there are no limitations in the host species of the primary antibody and cross-reactivity and no limitations with overlapping colors. 2) Tissue integrity. Tissue sections tolerate a high number of consecutive times in a pressure cooker (up to 8 rounds tested). However, detecting matrix proteins in desmoplastic or fibrotic tissues can be challenging. The order in which the primary antibodies are used needs to be optimized. For instance, it is advisable to use antibodies that recognize matrix-related proteins in the two first rounds. 3) Antigen retrieval. Other antigen retrieval methods have been tested such as microwave, water bath and enzyme digestion. The pressure cooker performs better than these methods because it can reach high-temperatures in short periods of time maintaining tissue integrity for further rounds. However, enzyme-based antigen retrieval such as pepsin, proteinase K, does not tolerate the “stripping and re-probing” method. Other antigens may also be affected using this method and giving negative results in the following rounds. 4) Montage and Image analysis. Image alignment using the proposed method is very laborious, especially if the user is working with high number of samples. Also, automation is limited and only possible for a few steps (see section automation and batch analysis).

## Troubleshooting

### Problem 1

Presence of different intensity patterns/intensity in Immunohistochemical staining.

### Potential solution

Changing from DAB to VIP chromogen may lead to variation in the intensity. When using an antibody for the first time, include positive and negative controls and parallel staining with DAB. Antibody concentrations need to be carefully titrated.

### Problem 2

Chromogen remaining from previous round after HIAR.

### Potential solution

Presence of chromogen (VIP) after HIAR in consecutive rounds might be due to suboptimal antigen retrieval or suboptimal optimization of previous round. Standard time for HIAR (10 min) needs to be prolonged for some antigens. Too intense staining due to saturation with primary antibody or exposure time with chromogen will affect the stripping process. Background-to-signal ratio needs optimization prior to cyclic IHC.

### Problem 3

Lack of staining in consecutive rounds.

### Potential solution

Tissues are sensitive to rounds of heat-induced antigen retrieval. Some antigens may no longer be detected. This is one the main benefits of using a pressure cooker since heat can be applied for very short periods of time. Soft-tissues or tissues rich in extracellular matrix (ECM) are more sensitive.

When using a panel of different antibodies, antibody order needs to be optimized. Very robust antigens such as pan-cytokeratin, ki67 among others should be added in the latest rounds.

### Problem 4

Automatic image alignment not working.

### Potential solution

**Figure 6:**
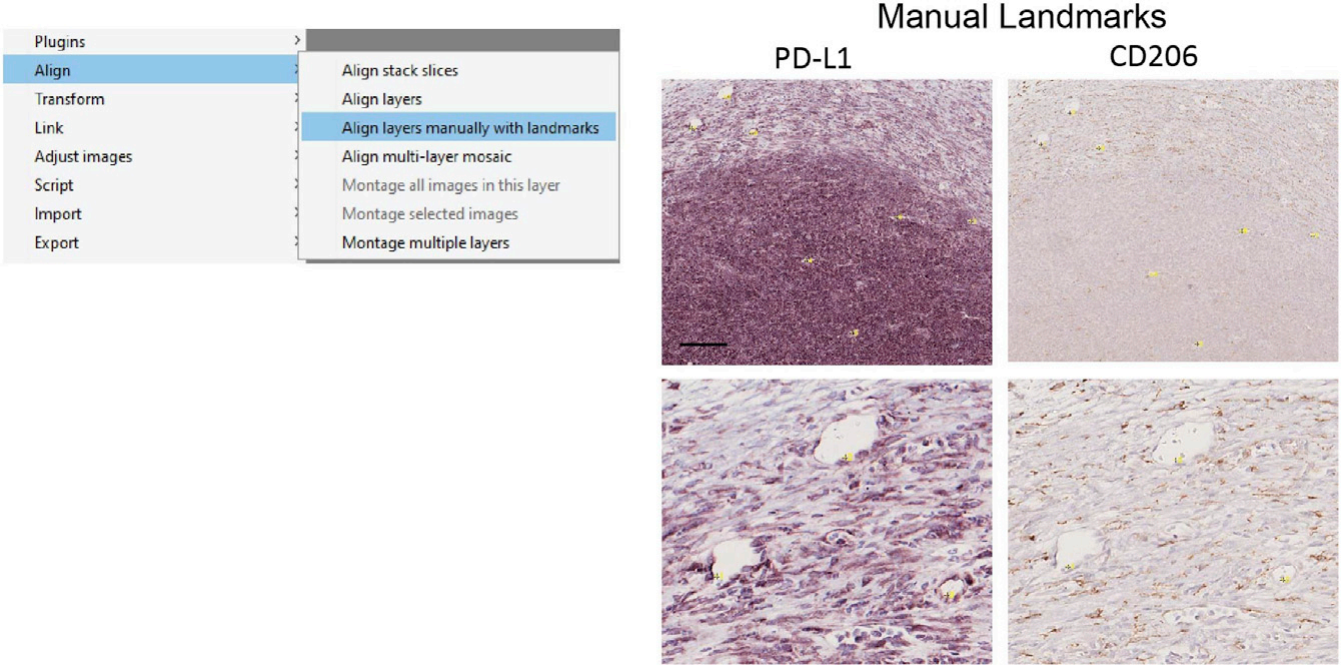
Representative screenshots and images showing how to perform manual alignment in TrackEM2 in Fiji ImageJ Scale bar: 200 μm.

TrackEM2 in FIJI-ImageJ also allows for supervised image alignment. Press “right click” in the mouse, go to “align”>”Align layers manually with landmarks”. As shown in Figure 6, landmarks by similarity need to be created in the consecutive images. Important: same mark needs to be clearly labelled for both sections (see Figure 6).

### Problem 5

Color deconvolution adjustment. Color deconvolution setting may need changes if the chromogens are not properly separated.

### Potential solution

Here we show a step-by-step process using ImageJ to set up a custom color deconvolution using a control ROI from same experiment as template. Instead of selecting H AEC or H DAB, we select from ROI in the color deconvolution window. Follow ImageJ instructions and create a square where the chromogen is present, it will be Color 1. Create a second square where Chromogen 2 is present (this case nucleus, blue). For Color 3 just press 3 “right click” if your image has two colors like Hematoxylin and VIP or DAB.

**Figure 7:**
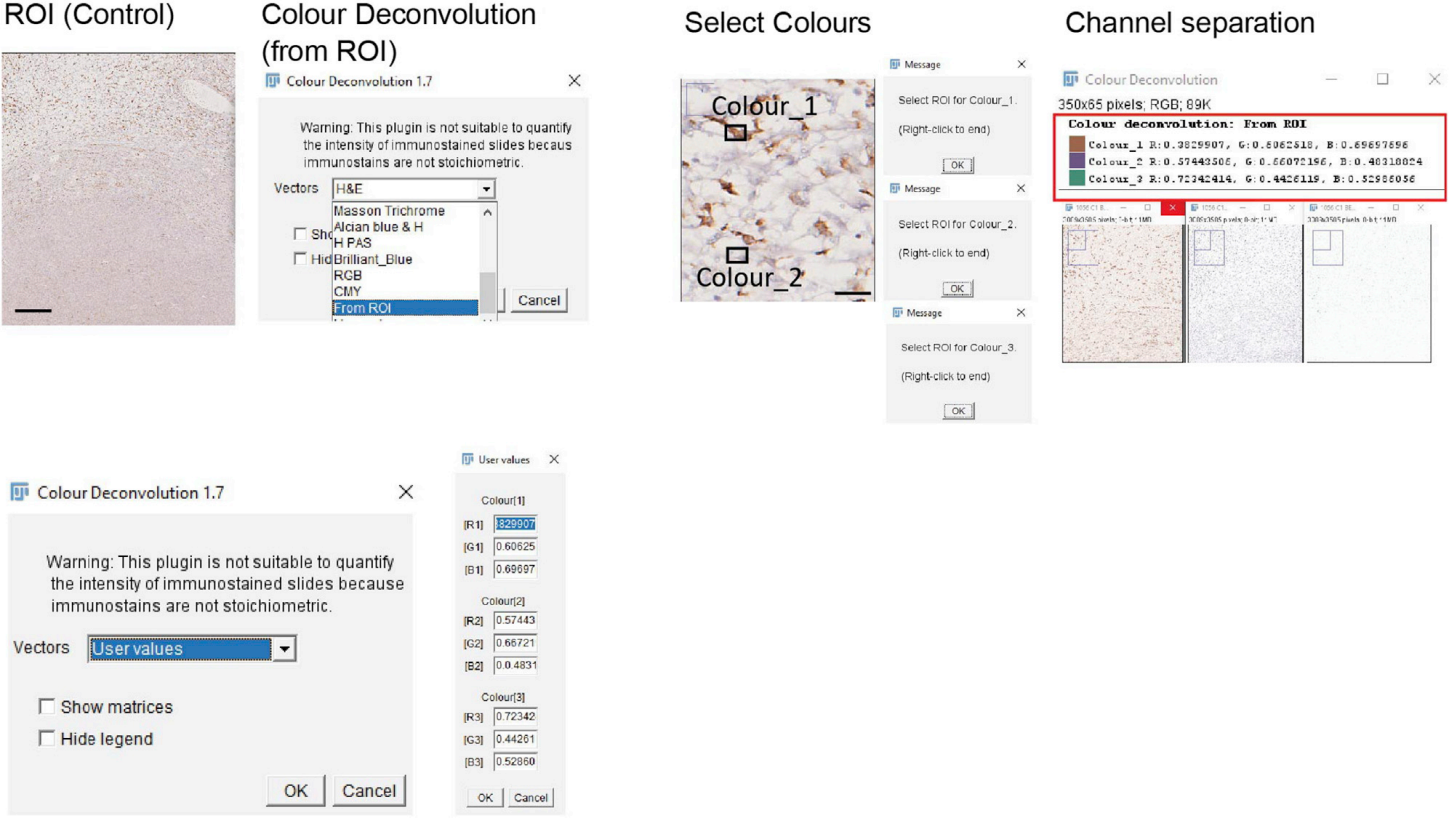
Representative screenshots and workflow for a step-by-step color deconvolution using custom chromogens Scale bars: 200 μm (left), 50 μm (right).

To apply the same setting in the following images from same experiment, go to color deconvolution and select User Values. Fill with the values obtained from control image (highlighted in red) in Figure 7.

### Problem 6

Cell segmentation issues.

### Potential solution

**Figure 8:**
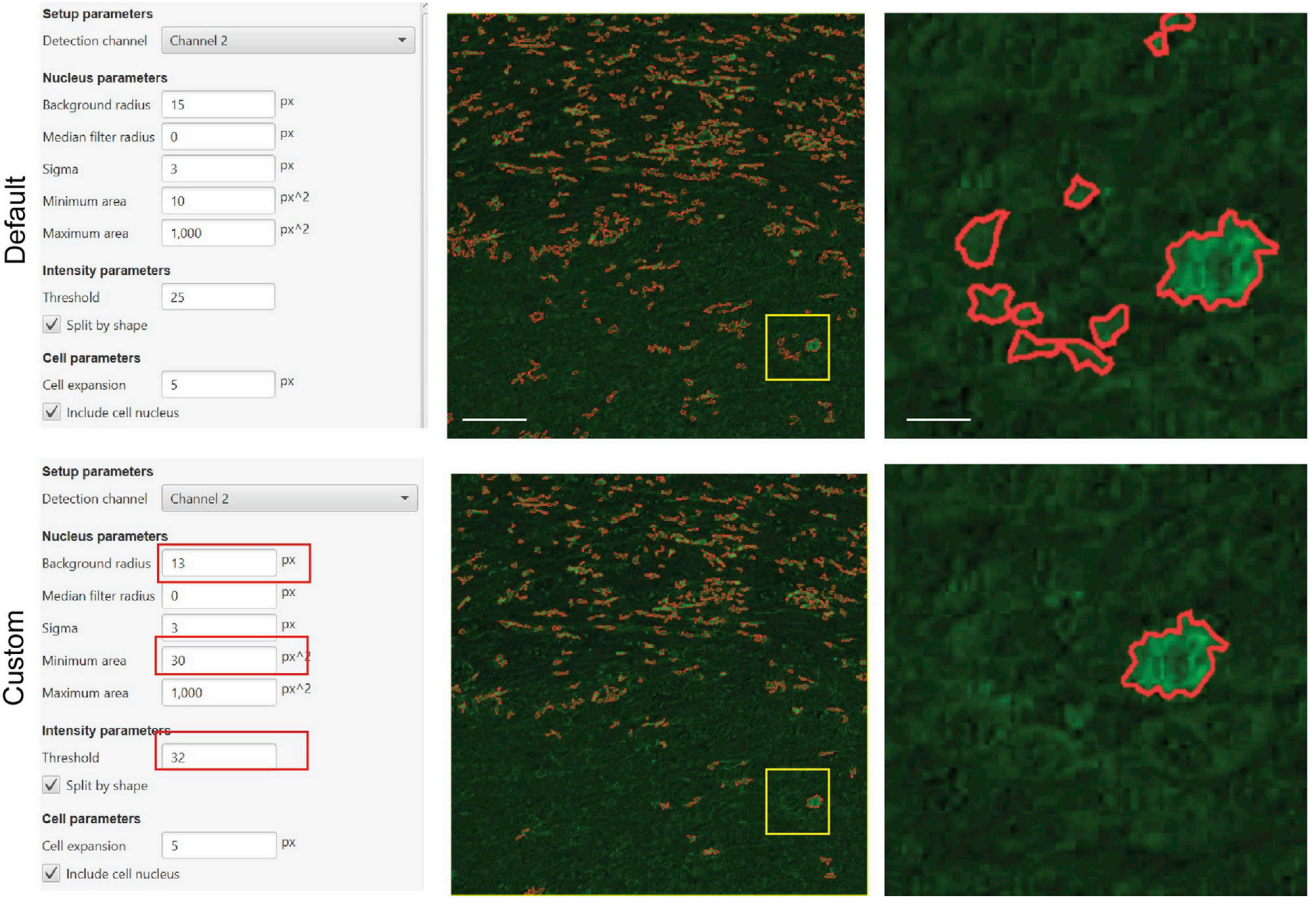
Representative images of Cell detection in Qupath (Left) Screenshots of Cell detection tab, red squares highlight changes performed. (Middle) Representative images in green channel (CD206) after cell segmentation in Default and custom setting. Scale bar: 100 μm. (Right) Magnification of yellow box from middle images. Scale bar: 10 μm.

The user may need to change some parameters according to the tissue or to cell composition. Important nuclear parameters are “Background radius”, “Median filter radius”, and “sigma”. Background radius subtracts a background value from every pixel on the image and after applies a threshold. In case of many nuclei clustered together, increasing background radius number aids correct nuclei segmentation. Median filter radius and sigma can be useful parameters to remove fragmented nuclei from analysis (see Figure 8). In our case, default settings were amended with some custom values to allow optimal cell segmentation (Figure 8).

## Resource availability

### Lead contact

Further information and requests for resources and reagents should be directed to and will be fulfilled by the lead contact, Prof Victoria Sanz-Moreno (v.sanz-moreno@qmul.ac.uk).

### Materials availability

No materials were newly generated for this protocol. All materials mentioned above are commercially available.

## Data Availability

Dataset used were published in JL Orgaz., et al. Cancer Cell 2020.^1^ Codes used are posted in section above “image analysis and quantification”.
